# The association between air pollutants and mild cognitive impairment in Taiwanese elderly

**DOI:** 10.7150/ijms.129021

**Published:** 2026-05-01

**Authors:** Sui-Lung Su, Yue-Ting Lin, Chih-Hong Pan, Meng-Chang Lee, Yu-Hsuan Chen, Hsiao-Ting Lin, Hao Su, Wei-Teing Chen

**Affiliations:** 1 Graduate Institute of Public Health, College of Public Health, National Defense Medical University, Taipei, Taiwan.; 2 Institute of Labor, Occupational Safety and Health, Ministry of Labor, New Taipei City, Taiwan.; 3 School of Public Health, College of Public Health, National Defense Medical University, Taipei, Taiwan.; 4 Graduate Institute of Medical Sciences, College of Medicine, National Defense Medical University, Taipei, Taiwan.; 5 Division of Thoracic Medicine, Department of Medicine, Cheng Hsin General Hospital, Taipei, Taiwan.; 6 Division of Thoracic Medicine, Department of Medicine, Tri-Service General Hospital, National Defense Medical University, Taipei, Taiwan.; 7 School of Medicine, College of Medicine, National Defense Medical University, Taipei, Taiwan.

**Keywords:** mild cognitive impairment (MCI), air pollutants, mini-mental state examination (MMSE)

## Abstract

**Background:**

Mild cognitive impairment (MCI) is considered a prodromal stage of dementia, and cognitive decline is generally challenging to reverse. Recently, air pollution has been identified as a risk factor for cognitive impairment, making the exploration of this association an essential research topic. However, nationwide studies investigating the relationship between air pollution and MCI are still lacking.

**Objective:**

This study aimed to investigate the association between air pollution and MCI in Taiwan.

**Methods:**

This cross-sectional study utilized data from the Taiwan Biobank, retrieved elderly participants aged above 60, between 2008 and 2021. The study collected demographic data and cognitive function assessments using the Mini-Mental State Examination (MMSE) scale. MCI was defined by cut-off scores of 18, 21, and 25 according to educational levels. Google Map was used to locate the nearest air quality monitoring station from the residential address of participants. Individual daily average exposure concentrations of PM_2.5_, PM_10_, SO_2_, O_3_, CO, and NO_2_ were sourced from the air quality monitoring station of Environmental Protection Administration (EPA) between 1993 and 2021. Regression models were employed to identify factors associated with MCI in Taiwan.

**Results:**

This study contains 4,173 cases with MCI and 31,022 controls, representing an MCI prevalence of 11.9%. The individual daily average exposure concentrations of PM_2.5_, PM_10_, O_3_, CO, SO_2_ and NO_2_ are 30.70±8.38 μg/m^3^, 59.25±15.81 μg/m^3^, 25.96±3.08 ppb, 0.65±0.42 ppm, 4.97±2.59 ppb and 20.22±5.09 ppb, respectively. Tertiles of pollution exposure in regression models show that higher exposure to PM_2.5_ and SO_2_ are associated with a higher risk of MCI compared to that of lower exposure levels (OR = 1.23, 95% CI: 1.11-1.37; OR = 1.20, 95% CI: 1.07-1.34). Furthermore, concurrent exposure to the highest tertiles (T3) of both PM_2.5_ and SO_2_ increased the risk of MCI to 2.33 (95% CI: 1.56-3.48).

**Conclusions:**

We found evidence of the effect of PM_2.5_ and SO_2_ on MCI among elderly individuals in Taiwan. Moreover, the association between PM_2.5_ and MCI risk escalated with higher SO_2_ concentrations, demonstrating a synergistic interaction between the two pollutants. This discovery may provide evidence for policy of MCI prevention.

## Introduction

With the rapid aging of the global population, the proportion of elderly people is expected to increase from 12% to 22% 1. In Taiwan, the elderly population is projected to reach 39.3% by 2060, making it the second-highest in the world 2. Research shows that over 20% of the elderly suffer from neurological diseases, with dementia and cognitive impairment being the most common among them 1. A previous survey in Taiwan (2011-2013) reported that the age- and sex-adjusted prevalence of overall dementia and mild cognitive impairment (MCI) in older adults were 8.13% and 18.78%, respectively 3, which is higher than the prevalence observed in East Asia (4.98-6.99% in 2015) 4.

Dementia is a neurodegenerative disease that severely impacts an individual's ability to perform daily activities independently, causing a heavy burden on caregivers 5, 6. MCI is considered a precursor to dementia, with about 5-10% of MCI individuals progressing to dementia each year 7, 8. As dementia is currently incurable and cognitive decline is difficult to reverse, identifying risk factors for MCI is crucial. The risk factors for cognitive decline include age, gender, education level, medical history, genetics, lifestyle, and environmental factors 9-12. Among these, the relationship between air pollutants and MCI is an important issue in public health. Air pollutants such as particulate matter (PM), nitrogen oxides, and ozone are powerful oxidants that can reach the brain, causing oxidative stress and neuroinflammation, which can affect the central nervous system 13, 14. Previous studies have shown that long-term exposure to particulate pollutants can lead to amyloid beta deposition in the brain, and exposure to PM_2.5_ and PM_10_ increases the risk of developing MCI 15, 16. Moreover, studies have also found that exposure to sulfur dioxide (SO_2_) causes synaptic dysfunction in the hippocampus, impairing memory in animal models 17. Since nationwide studies investigating the relationship between air pollution and MCI are still lacking, this study aimed to examine the association between air pollution and MCI in Taiwan.

## Materials and Methods

### Study population

**Figure 1:**
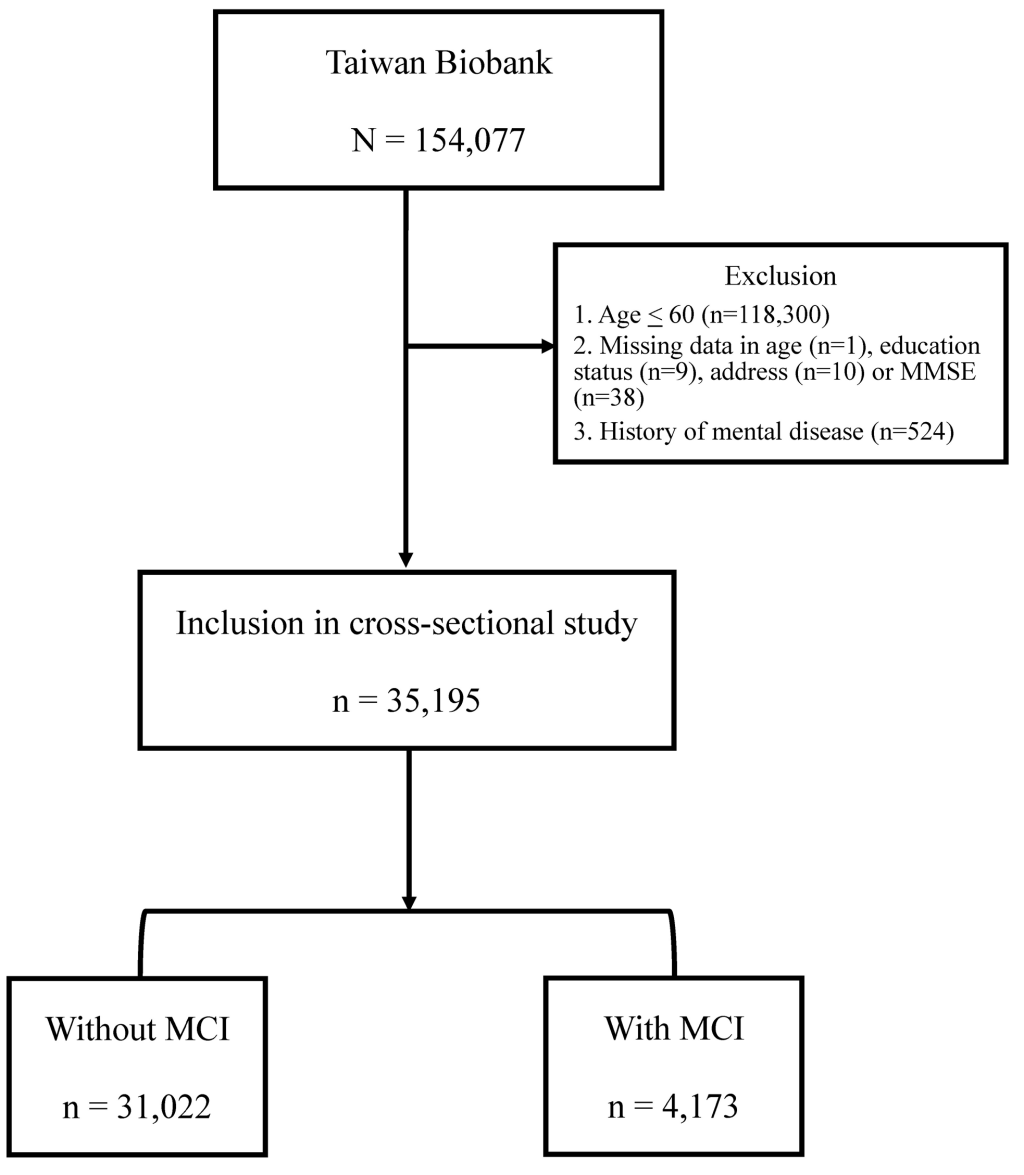
** Flow chart.** A total of 154,077 participants were analyzed using data from the TWB database. The exclusion criteria are individuals below the age of 60 (n = 118,300), those with incomplete information regarding age (n=1), education status (n=9), address (n=10) or MMSE (n=38), and history of mental disease (n = 524). The 35,195 subjects were recruited in the study, including of 4,173 subjects with MCI and 31,022 subjects without MCI.

The Taiwan Biobank (TWB) was established with the purpose of collecting genomic and lifestyle data from Taiwanese residents, and it is currently the largest biobank in Taiwan supported by the government 18. The TWB consists of data from community-based volunteers between the ages of 20 and 70. Prior to participation, all participants provided informed consent, donated blood samples, and underwent physical examinations. Various measurements such as body height, weight, and body mass index (BMI) (kg/m^2^) were recorded. Additionally, participants were interviewed face-to-face by TWB researchers and asked to complete questionnaires regarding personal and lifestyle factors. The data analysis for this study utilized the questionnaire survey data and relevant physical examination measurements of 154,077 participants from the Taiwan Biobank, collected between 2008 and 2021. After excluding individuals below the age of 60 or history of mental disease, as well as those with incomplete information regarding age, place of residence, Mini-Mental State Examination (MMSE), and education level, a total of 35,195 participants was included in the study. The flow chart was shown in Figure 1.

### Collection of demographic and medical data

Basic information about subjects were retrieved from TWB questionnaire, containing age, gender, education level, marital status, monthly income, residence, BMI, exercise habits, smoking, alcohol, hypertension, stroke, diabetes and depression.

### Assessment of mild cognitive impairment

Cognitive impairment was assessed by MMSE, including six domains: visuospatial, language, concentration, working memory, memory recall, and orientation 19. The scores of MMSE ranges from 0 to 30, and higher scores indicate better cognitive function. The cut-off scores for MCI were defined as scores ≤ 18 for illiterates, ≤ 21 for participants with primary school education, and ≤ 25 for those with junior high school degree or above 20. The 35,195 subjects were recruited in the study, including of 4,173 subjects with MCI and 31,022 subjects without MCI.

### Assessment of air pollutants

**Figure 2:**
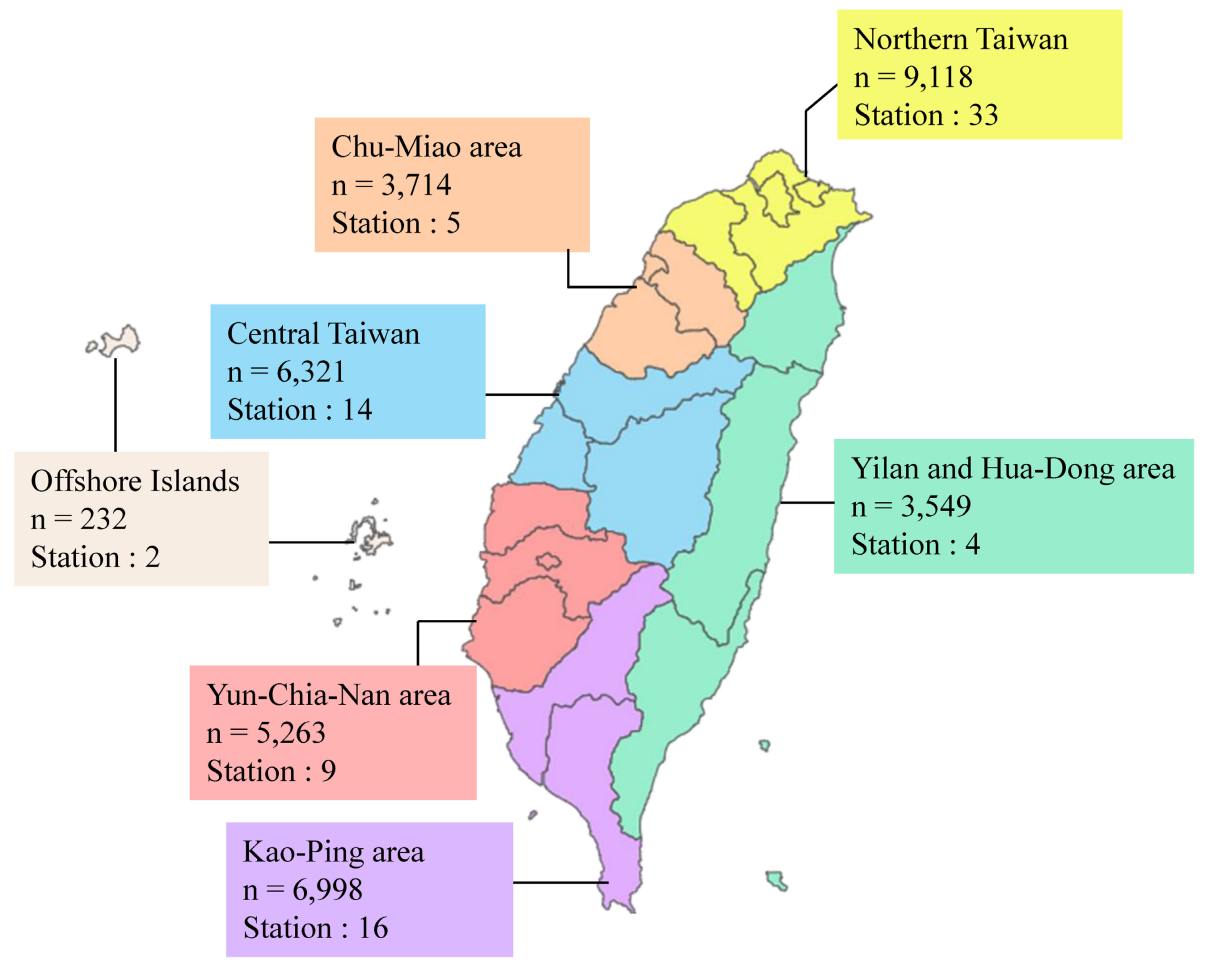
** Participant numbers and monitoring station distribution map for air quality zones in Taiwan.** Northern Taiwan includes a population of 9,118 individuals with 33 monitoring stations. The Chu-Miao area has a population of 3,714 individuals and 5 monitoring stations. Central Taiwan includes a population of 6,321 individuals with 14 monitoring stations. The Yun-Chia-Nan area has a population of 5,263 individuals and 9 monitoring stations. The Kao-Ping area has a population of 6,998 individuals with 16 monitoring stations. The Yilan and Hua-Dong area includes a population of 3,549 individuals with 4 monitoring stations. Lastly, the Offshore Islands include a population of 232 individuals and 2 monitoring stations.

This study collected air pollution concentration data from 83 monitoring stations provided by the Environmental Protection Administration (EPA) and the Environmental Protection Bureaus of five cities, including Taipei City, New Taipei City, Taoyuan City, Taichung City, and Kaohsiung City 21-26. Seven Air Quality Zones (AQZs) classified by the EPA: northern Taiwan (NT), the Chu-Miao (CM) area, central Taiwan (CT), the Yun-Chia-Nan (YCN) area, the Kao-Ping (KP) area, the Hua-Dong (HD) area, Yilan (YI), and the Offshore Islands 21. The distribution of participants and monitoring stations across these AQZs is shown in Figure 2.

The residential address of each participant was used to estimate exposure to outdoor air pollution. The average concentrations of air pollutants, including particulate matter with an aerodynamic diameter of ≤ 2.5 μm (PM_2.5_), particulate matter with an aerodynamic diameter of ≤ 10 μm (PM_10_), ozone (O_3_), carbon monoxide (CO), sulfur dioxide (SO_2_), and nitrogen dioxide (NO_2_) were calculated for each year. Average yearly data were determined in two steps: (1) obtaining the geographic coordinates (latitude and longitude) of the administrative district center of each participant's residential area and the monitoring station addresses from government open data platforms, the EPA, and the Environmental Protection Bureaus, and (2) using Google Maps to locate the nearest air quality monitoring station to each participant's residential administrative district as the data source. The participant's yearly average exposure concentrations of PM_10_, SO_2_, O_3_, NO_2_, CO were estimated from 1993 to the year of MMSE assessment. Since PM_2.5_ data were unavailable until 2005, the participant's yearly average exposure concentrations of PM_2.5_ was estimated from 2005 to the year of MMSE assessment 21.

### Statistical analysis

Data were expressed as mean ± standard deviation or frequency (%) and tertile for air pollutants concentration. Differences between groups were analyzed using the chi-square test for categorical variables and the independent t test for continuous variables. Logistic regression analysis was used to identify associations between each air pollutant and MMSE through three adjustment models. Model 1 was adjusted for gender, age, stroke, monthly income, BMI, exercise habits, marital status, smoking, alcohol, hypertension, and diabetes. Education level was excluded as a covariate because it was used to define MMSE cut-off scores, preventing over-adjustment bias. Model 2 was a multi-pollutant model that further adjusted for co-pollutants to account for the simultaneous exposure to multiple air pollutants. Model 3 further incorporated an interaction term between PM_2.5_ and SO_2_ (PM_2.5_ × SO_2_) into the multi-pollutant model to evaluate potential synergistic effects on the risk of MCI (Table S2). Furthermore, a sensitivity analysis was conducted by including depression as an additional covariate in the final logistic regression models to evaluate the robustness of our findings. This study considered a p value of < 0.05 as significant for all analyses. All analyses were performed using SPSS 25.0.

### Ethics statement

The study protocol followed the guidelines for human studies and was conducted ethically in accordance with the World Medical Association Declaration of Helsinki. The study was approved by the Institutional Review Board of the Tri-Service General Hospital (TSGH), a medical teaching hospital of the National Defense Medical University in Taipei, Taiwan (approval number: TSGH-2-107-05-091, approval date: 29 June 2018).

## Results

### Demographic data

The demographic characteristics of the study participants are shown in Table 1. The study included a total of 35,195 individuals, 39% of whom are male. The overall mean age is 64.19 ± 3.22 years old. Among participants without mild cognitive impairment (MCI) are 64.14 ± 3.17 years old. In contrast, participants with MCI exhibited a slightly higher mean age of 64.58 ± 3.54 years old. Significant differences are observed between the MCI and without MCI groups. The MCI group is characterized by older age (p < 0.001), lower educational level (p < 0.001), less married (p < 0.001), lower income (p < 0.001), higher body mass index (BMI) (p < 0.001), less exercise habits (p < 0.001), more smoking (p = 0.008), hypertension (p < 0.001), stroke (p < 0.001), and diabetes (p < 0.001).

### Estimation of air pollutants concentration among subjects with or without MCI

The estimation of air pollutants concentration among subjects with or without MCI are shown in Table 2. Participants with MCI are exposed to higher concentrations of PM_2.5_, PM_10_, and SO_2_, with mean levels of 31.30 ± 8.85 μg/m³, 59.98 ± 16.2 μg/m³, and 5.09 ± 2.71 ppb, respectively. The second tertiles (T2) or third tertiles (T3) have higher proportion of participants with MCI among PM_2.5_, PM_10_, O_3_, SO_2_, and NO_2_.

### Distribution of demographic data according to tertile of estimation of air pollutants concentration

The distribution of demographic characteristics across tertiles of PM_2.5_, PM_10_, O_3_, SO_2_, CO, and NO_2_ concentrations are shown in Table 3. Significant differences in variables such as gender, age, stroke, monthly income, BMI, exercise habits, marital/cohabitation status, smoking, alcohol consumption, hypertension, and diabetes were observed among the three groups.

### The association between tertile of air pollutants concentration and MCI

**Figure 3:**
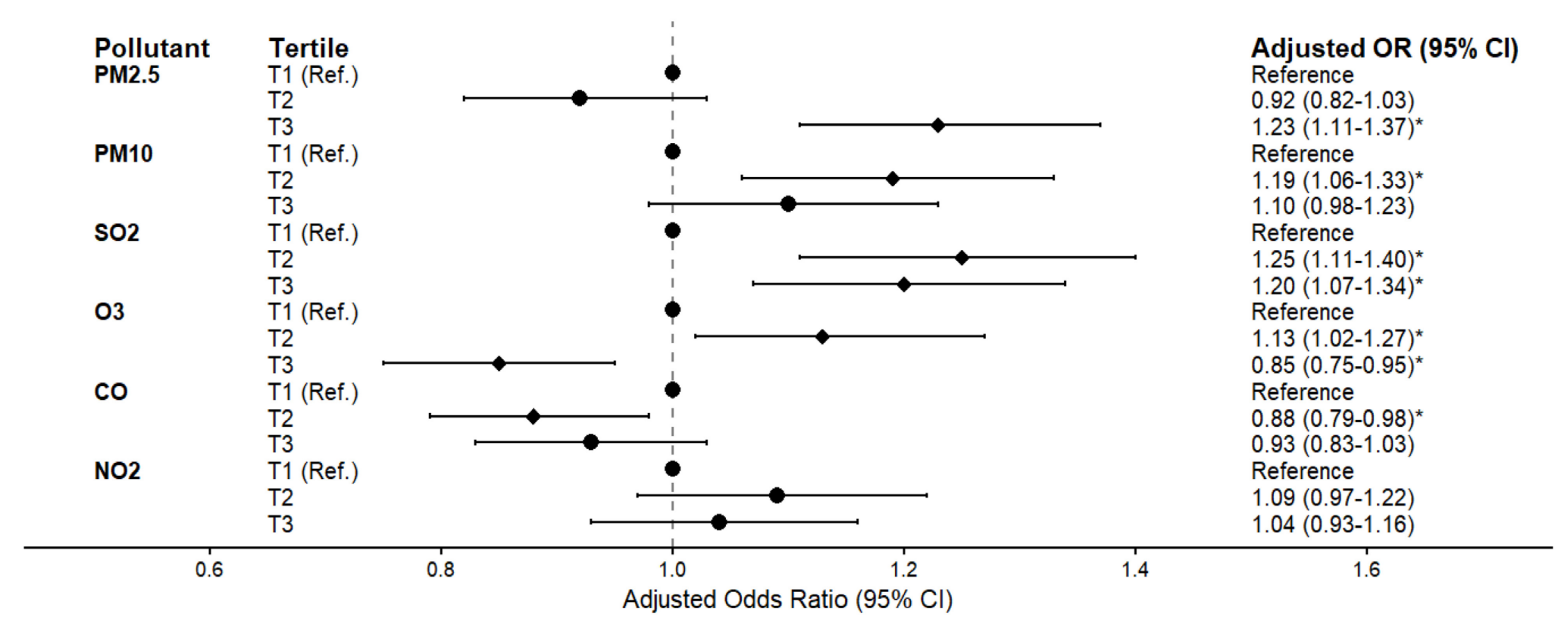
** The association between tertile of air pollutants concentration and MCI.** The forest plot displays the adjusted odds ratios (ORs) and 95% confidence intervals (CIs) for MCI across tertiles (T1, T2, and T3) of different air pollutants (PM_2.5_, PM_10_, SO_2_, O_3_, CO, and NO_2_), with the lowest tertile (T1) serving as the reference group. Data were adjusted for gender, age, stroke history, economic status, BMI, exercise habits, marital status, smoking, alcohol consumption, hypertension, and diabetes. The dots represent the ORs, and the horizontal bars represent the 95% CIs. *: *p* < 0.05.

**Figure 4:**
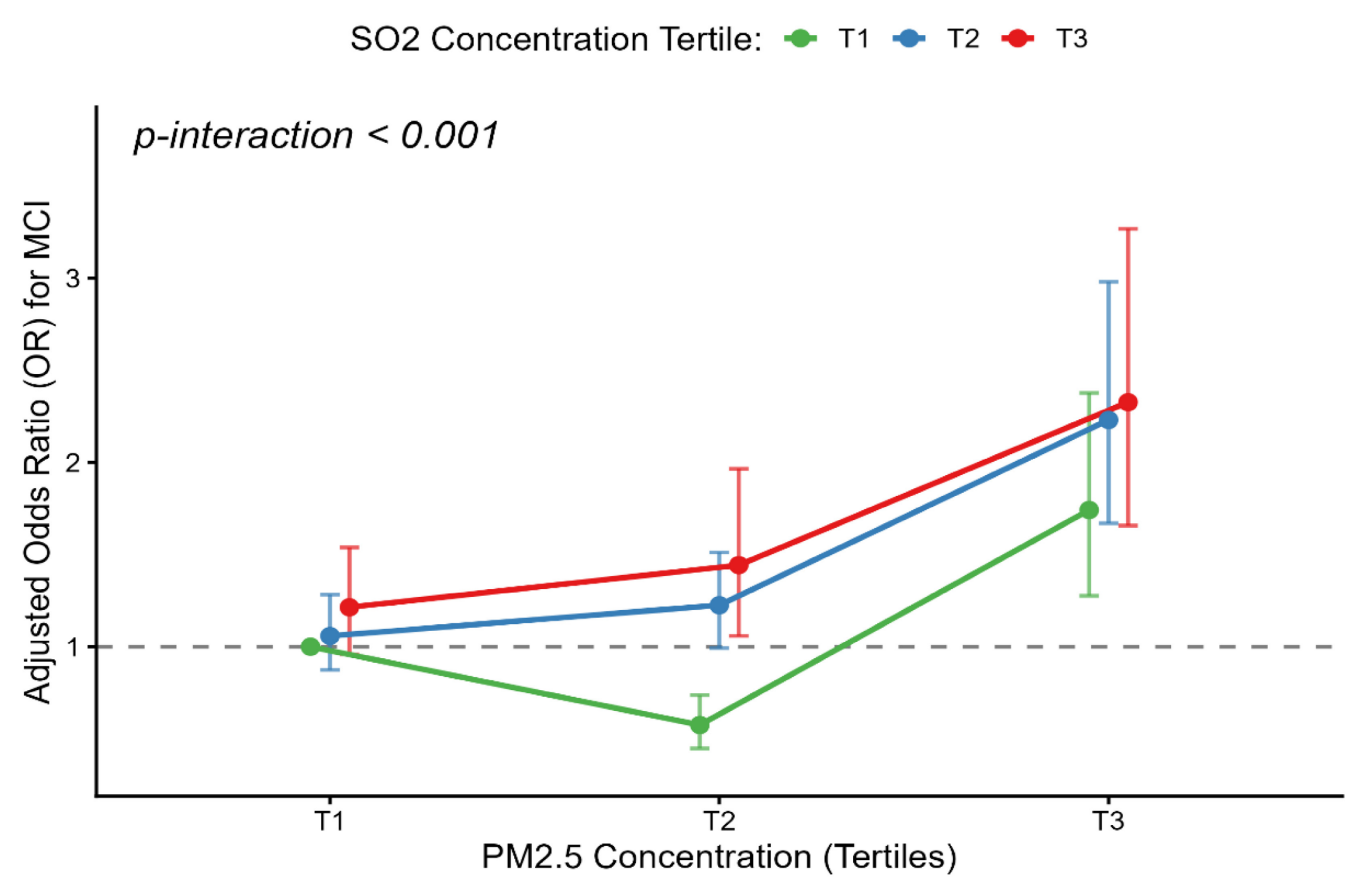
** Synergistic interaction of PM_2.5_ and SO_2_ on the risk of mild cognitive impairment.** The interaction plot displays the adjusted odds ratios (ORs) for Mild Cognitive Impairment (MCI) across different combinations of PM2.5 and SO2 tertiles, with the lowest co-exposure group (PM2.5 T1 and SO2 T1) serving as the reference. The X-axis represents the tertiles of PM2.5 concentration, and the Y-axis indicates the adjusted ORs for MCI. The green, blue, and red lines correspond to the first (T1), second (T2), and third (T3) tertiles of SO2, respectively. Data were adjusted for gender, age, stroke history, economic status, BMI, exercise habits, marital status, smoking, alcohol consumption, hypertension, and diabetes. The dots represent the specific adjusted ORs for each combination, and the vertical bars represent the 95% CIs.

The associations between tertile of air pollutants and the risk of MCI are presented in Figure 3 and Table S2. After adjusting for gender, age, stroke, economic status, BMI, exercise habits, marital status, smoking, alcohol consumption, hypertension, and diabetes, participants with second tertile (T2) groups exposure concentrations of PM_10_, SO_2_ and O_3_ had a significantly higher risk of MCI compared to those in the first tertile (T1) groups (PM_10_: OR = 1.19, 95% CI: 1.06-1.33; SO_2_: OR = 1.25, 95% CI: 1.11-1.40; O_3_: OR = 1.13, 95% CI: 1.02-1.27). Meanwhile, participants with third tertile (T3) groups exposure concentrations of PM_2.5_ and SO_2_ had similar trends (PM_2.5_: OR = 1.23, 95% CI: 1.11-1.37; SO_2_: OR = 1.20, 95% CI: 1.07-1.34). However, participants exposed to higher concentrations of O_3_ and CO had a significantly lower risk of MCI compared to those in T1 groups (O_3_: OR = 0.85, 95% CI: 0.75-0.95; CO: OR = 0.88, 95% CI: 0.79-0.98). Meanwhile, the exposure concentrations of NO_2_ were not associated with MCI (OR = 1.04, 95% CI: 0.93 - 1.16). Furthermore, a highly significant interaction between PM_2.5_ and SO_2_ (PM_2.5_ × SO_2_) was observed (*p*-interaction < 0.001). After adjusting for co-pollutants and this interaction (Table S2), the independent effect of SO_2_ became non-significant, while the highest PM_2.5_ exposure (T3) remained robustly associated with MCI (OR = 1.74, 95% CI: 1.27-2.37). Crucially, concurrent exposure to the highest tertiles (T3) of both pollutants amplified the risk (OR = 2.33, 95% CI: 1.56-3.48) (Figure 4). Conversely, moderate PM_2.5_ (T2) showed a negative association (OR = 0.58, 95% CI: 0.45-0.74) compared to the reference group. Additionally, a sensitivity analysis was performed by further adjusting for depression in the final model (Table S3). The primary associations between air pollutants and the risk of MCI remained fundamentally unchanged, confirming the stability and reliability of our findings.

## Discussion

There are 4,173 cases of MCI and 31,022 control participants, with an overall prevalence of MCI of 11.9%. The exposure to air pollutants concentrations were as followed: PM_2.5_ (30.70 ± 8.38 μg/m³), PM_10_ (59.25 ± 15.81 μg/m³), O₃ (25.96 ± 3.08 ppb), CO (0.65 ± 0.42 ppm), SO₂ (4.97 ± 2.59 ppb), and NO₂ (20.22 ± 5.09 ppb). Tertiles of air pollutants exposure reveals those higher levels of exposure to PM_2.5_ and SO₂ are significantly associated with an increased risk of MCI, with OR of 1.23 (95% CI: 1.11-1.37) and 1.20 (95% CI: 1.07-1.34), respectively, when compared to that of lower exposure levels.

A Swedish study revealed that elderly individuals exposed to high levels of PM experienced significant cognitive decline 27. Similarly, research conducted in the United States found that PM_2.5_ exposure negatively affected cognitive function in older women 28. In Taiwan, Chen et al. reported that long-term exposure to PM_2.5_ were associated with poor cognitive function in Taiwanese community-dwelling older adults 29, 30. PM has been recognized as a major factor contributing to Alzheimer's disease 31, 32. Although PM_2.5_ and PM_10_ share neurodegenerative mechanisms 33-35, the apparent protective effect of PM_10_ in Model 2 is likely a statistical artifact of multicollinearity (Beta = 0.87, p < 0.001) rather than a true biological benefit 36.

In this study, Higher SO₂ exposure associated with higher risk of MCI. Research by Park et al. and Chen et al. demonstrated that higher SO₂ exposure levels were correlated with lower MMSE scores, particularly in the domains of orientation, recall, and language abilities 30, 37. Additionally, animal studies have confirmed that SO₂ exposure leads to increased pro-inflammatory cytokine levels, the production of amyloid-β protein in the brain, and hippocampal synaptic dysfunction, leading to cognitive impairment 17, 38, 39. Notably, our interaction analysis reveals a significant interaction between PM_2.5_ and SO₂. The negative association between moderate PM_2.5_ (T2) and MCI may be consistent with the concept of hormesis 40, where mild environmental stress activates adaptive neuroprotective pathways 41. Conversely, high PM_2.5_ exposure (T3) combined with SO₂ is synergistically associated with a higher risk of MCI, a finding that aligns with experimental models showing neurodegeneration through apoptosis and synaptic damage 42. Our findings suggest that SO₂ may act as a critical effect modifier, elevating the risk of MCI during co-exposure with PM_2.5_.

Chen et al., using data from the Taiwan Biobank and Environmental Protection Administration, observed that higher O₃ concentrations had a protective effect on cognitive function 30. Likewise, increase O₃ concentrations were associated with better cognition of long-term exposure in Taiwanese community-dwelling older adults 43. In Korea, Park et al. used MMSE to assess cognitive function in adults aged 60 and above, finding that higher O₃ concentrations were associated with higher MMSE scores 37, 44. These findings are consistent with the results of the present study. A potential biological explanation for this protective association is the "hormetic effect," where low-level oxidative stress induced by O_3_ may trigger adaptive cellular responses. This includes the activation of the Nrf2 signaling pathway and upregulation of endogenous antioxidant enzymes (e.g., superoxide dismutase), which collectively enhance neuroprotective capacity against cognitive decline 45, 46. However, a cohort study in China tracking 9,544 individuals aged 65 and older reported that for every 10-unit increase in average O₃ exposure, the risk of cognitive impairment rose by 10.4%, contradictory to our findings, probably due to the different criteria of MMSE cutoff score 47. O₃ can trigger the release of pro-inflammatory cytokines such as IL-1β, TNF-α, and IL-6 and disrupt the blood-brain barrier, leading to increased synthesis of inflammatory molecules in the brain 48, 49; however, Iaccarino et al. found that exposure to PM_2.5_ concentrations was associated with amyloid PET scan, but not O₃ 50, suggesting that future study should be conducted to explore the mechanism of O₃ and cognitive function.

Although many studies have identified that CO and NO_2_ might negatively affect cognitive function 51-53, our study showed that the tertile of NO_2_ concentrations were not associated with MCI. Further experimental and epidemiological studies are needed to confirm the causal relationships between NO_2_ and MCI. While CO exposure exhibited an inverse association with MCI, suggesting a potential protective effect. This phenomenon could be attributed to the anti-inflammatory and neuroprotective properties of low-dose CO 54.

This study has four limitations. First, it is a cross-sectional study, we could not examine the effects of long-term exposure to these air pollutants on cognitive functioning. Second, the study participants are volunteers from the Taiwan Biobank, who may have higher health literacy and better economic conditions and lifestyles compared to that of the general population. Third, air pollution exposure was calculated using only the data from the nearest monitoring administrative district centers, which may not precisely evaluate individual exposure levels. However, as 88% of the administrative centers are located within 20 km of a station, the potential misclassification bias is considered to be minimized (Figure S1). Finally, air pollution levels were retrieved from air quality monitoring stations, which serve as a crude measure of exposure. While outdoor air pollution significantly contributes to indoor and personal exposure, it may not accurately represent actual personal exposure levels.

## Conclusions

This study discloses that an effect of PM_2.5_ and SO_2_ on MCI among elderly individuals in Taiwan. Moreover, synergistic interaction exists between the two pollutants, with the risk of MCI significantly escalating as PM_2.5_ concentrations increase. This discovery may provide evidence for policy of MCI prevention. These findings can serve as a valuable reference for formulating air pollution control measures and dementia prevention policies in Taiwan, highlighting the importance of addressing environmental factors in the mitigation of MCI risk.

## Supplementary Material

Supplementary figure and tables.

## Figures and Tables

**Table 1:** Demographic data

Variable	Total (n=35,195)	Without MCI (n = 31,022)	With MCI (n = 4,173)	P-value
Male	13724 (39.0%)	12114 (39.0%)	1610 (38.6%)	0.560
Age	64.19±3.22	64.14±3.17	64.58±3.54	< 0.001*
60-75	34936 (99.3%)	30820 (99.3%)	4116 (98.6%)	< 0.001*
> 75	259 (0.7%)	202 (0.7%)	57 (1.4%)	
Education				< 0.001*
illiterate	247 (0.7%)	152 (0.5%)	95 (2.3%)	
primary school	5065 (14.4%)	4395 (14.2%)	670 (16.1%)	
junior high school or above	29883 (84.9%)	26475 (85.3%)	3408 (81.7%)	
Married/Cohabitant	27151 (77.2%)	24051 (77.6%)	3100 (74.4%)	< 0.001*
Monthly income >100,000 NTD	1510 (7.6%)	1414 (8.0%)	96 (4.3%)	< 0.001*
BMI				< 0.001*
< 18.5 kg/m^2^	819 (2.3%)	744 (2.4%)	75 (1.8%)	
18.5≤X < 24 kg/m^2^	16443 (46.7%)	14661 (47.3%)	1782 (42.8%)	
24≤X < 27 kg/m^2^	11034(31.4%)	9689 (31.2%)	1345 (32.3%)	
≥ 27 kg/m^2^	6879 (19.6%)	5916 (19.1%)	963 (23.1%)	
Exercise habits	16910 (48.1%)	15047 (48.5%)	1863 (44.6%)	< 0.001*
Smoking	4763 (14.4%)	4138 (14.2%)	625 (15.8%)	0.008*
Alcohol	3249 (9.2%)	2851 (9.2%)	398 (9.6%)	0.459
Hypertension	8930 (25.4%)	7722 (24.9%)	1208 (28.9%)	< 0.001*
Stroke	492 (1.4%)	398 (1.3%)	94 (2.3%)	< 0.001*
Diabetes	3938 (11.2%)	3376 (10.9%)	562 (13.5%)	< 0.001*
Depression	1301 (3.7%)	1137 (3.7%)	164 (3.9%)	0.395

*: P-value < 0.05

**Table 2:** Estimation of air pollutants concentration among subjects with or without MCI

Variable	Total (n = 35,195)	Without MCI (n = 31,022)	With MCI (n = 4,173)	P-value
Air pollutants				
PM_2.5_ (μg/ 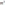 )	30.70±8.38	30.62±8.32	31.30±8.85	< 0.001*
PM_10_ (μg/ 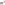 )	59.25±15.8	59.15±15.8	59.98±16.2	0.002*
O_3_ (ppb)	25.96±3.08	25.96±3.08	25.99±3.02	0.570
CO (ppm)	0.65±0.42	0.65±0.36	0.65±0.74	0.542
SO_2_ (ppb)	4.97±2.59	4.95±2.57	5.09±2.71	0.001*
NO_2_ (ppb)	20.22±5.09	20.25±5.09	20.04±5.05	0.014*
PM_2.5_ tertile				< 0.001*
T1 (≤ 26.53 μg/ 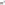 )	11730 (33.3%)	10394 (33.5%)	1336 (32.0%)	
T2 (26.54-34.86 μg/ 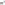 )	11729 (33.3%)	10448 (33.7%)	1281 (30.7%)	
T3 (> 34.87 μg/ 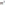 )	11736 (33.3%)	10180 (32.8%)	1556 (37.3%)	
PM_10_ tertile				< 0.001*
T1 (≤ 49.89 μg/ 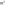 )	11731 (33.3%)	10476 (33.8%)	1255 (30.1%)	
T2 (49.9-68.3 μg/ 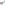 )	11730 (33.3%)	10242 (33.0%)	1488 (35.7%)	
T3 (> 68.31 μg/ 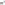 )	11734 (33.3%)	10304 (33.2%)	1430 (34.3%)	
O_3_ tertile				< 0.001*
T1 (≤ 24.59 ppb)	11729 (33.3%)	10361 (33.4%)	1368 (32.8%)	
T2 (24.6-27.19 ppb)	11732 (33.3%)	10144 (32.7%)	1588 (38.1%)	
T3 (> 27.2 ppb)	11734 (33.3%)	10517 (33.9%)	1217 (29.2%)	
CO tertile				0.001*
T1 (≤ 0.56 ppm)	11724 (33.3%)	10235 (33.0%)	1489 (35.7%)	
T2 (0.57-0.68 ppm)	11738 (33.4%)	10365 (33.4%)	1373 (32.9%)	
T3 (> 0.69 ppm)	11733 (33.3%)	10422 (33.6%)	1311 (31.4%)	
SO_2_ tertile				< 0.001*
T1 (≤ 3.79 ppb)	11729 (33.3%)	10472 (33.8%)	1257 (30.1%)	
T2 (3.8-5.49 ppb)	11727 (33.3%)	10268 (33.1%)	1459 (35.0%)	
T3 (> 5.5 ppb)	11739 (33.4%)	10282 (33.1%)	1457 (34.9%)	
NO_2_ tertile				0.020*
T1 (≤ 18.75 ppb)	11731 (33.3%)	10349 (33.4%)	1382 (33.1%)	
T2 (18.76-22.49 ppb)	11729 (33.3%)	10265 (33.1%)	1464 (35.1%)	
T3 (> 22.5 ppb)	11735 (33.3%)	10408 (33.6%)	1327 (31.8%)	

*: P-value<0.05

**Table 3:** Distribution of demographic data according to tertile of estimation of air pollutants concentration

Variable	PM_2.5_ (μg/m^3^)				PM_10_ (μg/m^3^)				O_3_ (ppb)			
	T1	T2	T3	P-value	T1	T2	T3	P-value	T1	T2	T3	P-value
Male	4554(38.8%)	4566(38.9%)	4604(39.2%)	0.803	4539(38.7%)	4563(38.9%)	4622(39.4%)	0.531	4427(37.7%)	4659(39.7%)	4638(39.5%)	0.003*
Age	64.46±3.50	64.15±3.11	63.96±3.00	<0.001*	64.34±3.42	64.19±3.15	64.03±3.06	<0.001*	64.25±3.28	64.14±3.20	64.17±3.18	0.028*
Education > 12 years	5166(44.0%)	4819(41.1%)	4694(40.0%)	<0.001*	5199(44.3%)	4689(40.0%)	4791(40.8%)	<0.001*	6577(56.1%)	7142(60.9%)	6797(57.9%)	< 0.001*
Married/Cohabitant	8878(75.7%)	9055(77.3%)	9218(78.6%)	<0.001*	8912(76.0%)	9064(77.3%)	9175(78.2%)	<0.001*	8907(76.1%)	9073(77.4%)	9171(78.2%)	< 0.001*
Monthly income > 100,000 NTD	714(8.9%)	431(7.5%)	365(6.1%)	<0.001*	644(9.4%)	464(7.3%)	402(6.1%)	<0.001*	529(8.3%)	514(7.7%)	467(6.9%)	0.009*
BMI				0.183				0.076				0.001*
< 18.5 kg/m^2^	265(2.3%)	270(2.3%)	284(2.4%)		273(2.3%)	262(2.2%)	284(2.4%)		280(2.4%)	274(2.3%)	265(2.3%)	
18.5≤X < 24 kg/m^2^	5459(46.6%)	5449(46.5%)	5535(47.2%)		5502(46.9%)	5395(46.0%)	5546(47.3%)		5638(48.1%)	5334(45.5%)	5471(46.7%)	
24≤X < 27 kg/m^2^	3625(30.9%)	3704(31.6%)	3705(31.6%)		3591(30.6%)	3771(32.2%)	3672(31.3%)		3644(31.1%)	3721(31.7%)	3669(31.3%)	
≥ 27 kg/m^2^	2374(20.3%)	2299(19.6%)	2206(18.8%)		2359(20.1%)	2295(19.6%)	2225(19.0%)		2159(18.4%)	2398(20.4%)	2322(19.8%)	
Exercise habits	5456(46.5%)	5512(47.0%)	5942(50.6%)	<0.001*	5435(46.3%)	5595(47.7%)	5880(50.1%)	<0.001*	5736(48.9%)	5563(47.4%)	5611(47.8%)	0.060
Smoking	1620(14.7%)	1618(14.7%)	1525(13.7%)	0.055	1610(14.6%)	1636(14.9%)	1517(13.7%)	0.020*	1534(13.9%)	1638(14.9%)	1591(14.3%)	0.102
Alcohol	1132(9.7%)	1076(9.2%)	1041(8.9%)	0.119	1127(9.6%)	1094(9.3%)	1028(8.8%)	0.075	1013(8.6%)	1156(9.9%)	1080(9.2%)	0.006*
Hypertension	2932(25.0%)	2969(25.3%)	3029(25.8%)	0.353	2909(24.8%)	2985(25.4%)	3036(25.9%)	0.162	2920(24.9%)	3005(25.6%)	3005(25.6%)	0.347
Stroke	187(1.6%)	160(1.4%)	145(1.2%)	0.060	179(1.5%)	165(1.4%)	148(1.3%)	0.224	154(1.3%)	174(1.5%)	164(1.4%)	0.540
Diabetes	1297(11.1%)	1271(10.8%)	1370(11.7%)	0.108	1275(10.9%)	1273(10.9%)	1390(11.8%)	0.022*	1232(10.5%)	1351(11.5%)	1355(11.5%)	0.016*
Depression	450(3.8%)	413(3.5%)	438(3.7%)	0.428	425(3.6%)	432(3.7%)	444(3.8%)	0.804	428(3.6%)	464(4.0%)	409(3.5%)	0.154
Variable	SO_2_ (ppb)				CO(ppm)				NO_2_(ppb)			
	T1	T2	T3	P-value	T1	T2	T3	P-value	T1	T2	T3	P-value
Male	4583(39.1%)	4636(39.5%)	4505(38.4%)	0.188	4701(40.1%)	4597(39.2%)	4426(37.7%)	0.001*	4701(40.1%)	4640(39.6%)	4383(37.3%)	< 0.001*
Age	64.42±3.44	64.08±3.14	64.06±3.05	<0.001*	64.19±3.31	64.15±3.14	64.22±3.20	0.195	64.32±3.41	64.03±3.05	64.21±3.17	< 0.001*
Education > 12 years	5164(44.0%)	4468(38.1%)	5047(43.0%)	<0.001*	4382(37.4%)	4644(39.6%)	5653(48.2%)	<0.001*	4494(38.3%)	4522(38.6%)	5663(48.3%)	< 0.001*
Married/Cohabitant	8982(76.6%)	9146(78.1%)	9023(76.9%)	0.023*	9165(78.2%)	9045(77.1%)	8941(76.3%)	0.002*	9083(77.5%)	9161(78.2%)	8907(76.0%)	< 0.001*
Monthly income > 100,000 NTD	569(8.4%)	486(7.5%)	455(7.0%)	0.008*	507(7.5%)	430(6.7%)	573(8.6%)	<0.001*	540(7.2%)	397(6.9%)	573(8.7%)	< 0.001*
BMI				<0.001*				<0.001*				< 0.001*
< 18.5 kg/m^2^	249(2.1%)	256(2.2%)	314(2.7%)		224(1.9%)	275(2.3%)	320(2.7%)		225(1.9%)	277(2.4%)	317(2.7%)	
18.5≤X < 24 kg/m^2^	5475(46.7%)	5324(45.4%)	5644(48.1%)		5246(44.8%)	5438(46.4%)	5759(49.1%)		5255(44.8%)	5444(46.4%)	5744(49.0%)	
24≤X < 27 kg/m^2^	3672(31.3%)	3806(32.5%)	3556(30.3%)		3757(32.1%)	3688(31.4%)	3589(30.6%)		3746(32.0%)	3723(31.8%)	3565(30.4%)	
≥ 27 kg/m^2^	2329(19.9%)	2333(19.9%)	2217(18.9%)		2493(21.3%)	2326(19.8%)	2060(17.6%)		2498(21.3%)	2280(19.4%)	2101(17.9%)	
Exercise habits	5645(48.1%)	5421(46.2%)	5844(49.8%)	<0.001*	5425(46.3%)	5563(47.4%)	5922(50.5%)	<0.001*	5425(46.2%)	5707(48.7%)	5778(49.2%)	< 0.001*
Smoking	1613(14.6%)	1689(15.3%)	1461(13.3%)	<0.001*	1719(15.6%)	1562(14.1%)	1482(13.6%)	<0.001*	1682(15.2%)	1615(14.6%)	1466(13.4%)	< 0.001*
Alcohol	1128(9.6%)	1161(9.9%)	960(8.2%)	<0.001*	1204(10.3%)	1037(8.8%)	1008(8.6%)	<0.001*	1208(10.3%)	1079(9.2%)	962(8.2%)	< 0.001*
Hypertension	2956(25.2%)	3050(26.0%)	2924(24.9%)	0.134	3076(26.2%)	3004(25.6%)	2850(24.3%)	0.002*	3076(26.2%)	3031(25.8%)	2823(24.1%)	<0.001*
Stroke	177(1.5%)	161(1.4%)	154(1.3%)	0.420	171(1.5%)	172(1.5%)	149(1.3%)	0.351	192(1.6%)	153(1.3%)	147(1.3%)	0.025*
Diabetes	1253(10.7%)	1406(12.0%)	1279(10.9%)	0.003*	1344(11.5%)	1345(11.5%)	1249(10.6%)	0.073	1359(11.6%)	1354(11.5%)	1225(10.4%)	0.007*
Depression	415(3.5%)	445(3.8%)	441(3.8%)	0.532	404(3.4%)	439(3.7%)	458(3.9%)	0.170	405(3.5%)	438(3.7%)	458(3.9%)	0.181

*: P-value < 0.05

## Data Availability

The datasets used and analysed during the current study available from the corresponding author on reasonable request.
