# Supplementary material for: The association between air pollutants and mild cognitive impairment in Taiwanese elderly
Source: Int J Med Sci. 2026 May 1;23(6):2072–80. doi: 10.7150/ijms.129021 (PMC13181367; doi:10.7150/ijms.129021)
Supplement: Supplementary file 1 — Supplementary figure and tables. [file ijmsv23p2072s1.pdf]

Table S1. Distribution of demographic data according to tertile of estimation of air pollutants concentration

Variable	O_3(ppb)				CO(ppm)				NO_2(ppb)			
	T1	T2	T3	P-value	T1	T2	T3	P-value	T1	T2	T3	P-value
Male	4427(37.7%)	4659(39.7%)	4638(39.5%)	0.003*	4701(40.1%)	4597(39.2%)	4426(37.7%)	0.001*	4701(40.1%)	4640(39.6%)	4383(37.3%)	<0.001*
Age	64.25±3.28	64.14±3.20	64.17±3.18	0.028*	64.19±3.31	64.15±3.14	64.22±3.20	0.195	64.32±3.41	64.03±3.05	64.21±3.17	<0.001*
Education >12 years	6577(56.1%)	7142(60.9%)	6797(57.9%)	<0.001*	4382(37.4%)	4644(39.6%)	5653(48.2%)	<0.001*	4494(38.3%)	4522(38.6%)	5663(48.3%)	<0.001*
Married/Cohabitant	8907(76.1%)	9073(77.4%)	9171(78.2%)	<0.001*	9165(78.2%)	9045(77.1%)	8941(76.3%)	0.002*	9083(77.5%)	9161(78.2%)	8907(76.0%)	<0.001*
Monthly income >100,000 NTD	529(8.3%)	514(7.7%)	467(6.9%)	0.009*	507(7.5%)	430(6.7%)	573(8.6%)	<0.001*	540(7.2%)	397(6.9%)	573(8.7%)	<0.001*
BMI				0.001*				<0.001*				<0.001*
<18.5 kg/m^2	280(2.4%)	274(2.3%)	265(2.3%)		224(1.9%)	275(2.3%)	320(2.7%)		225(1.9%)	277(2.4%)	317(2.7%)	
18.5≤X<24 kg/m^2	5638(48.1%)	5334(45.5%)	5471(46.7%)		5246(44.8%)	5438(46.4%)	5759(49.1%)		5255(44.8%)	5444(46.4%)	5744(49.0%)	
24≤X<27 kg/m^2	3644(31.1%)	3721(31.7%)	3669(31.3%)		3757(32.1%)	3688(31.4%)	3589(30.6%)		3746(32.0%)	3723(31.8%)	3565(30.4%)	
≥27 kg/m^2	2159(18.4%)	2398(20.4%)	2322(19.8%)		2493(21.3%)	2326(19.8%)	2060(17.6%)		2498(21.3%)	2280(19.4%)	2101(17.9%)	
Exercise habits	5736(48.9%)	5563(47.4%)	5611(47.8%)	0.060	5425(46.3%)	5563(47.4%)	5922(50.5%)	<0.001*	5425(46.2%)	5707(48.7%)	5778(49.2%)	<0.001*
Smoking	1534(13.9%)	1638(14.9%)	1591(14.3%)	0.102	1719(15.6%)	1562(14.1%)	1482(13.6%)	<0.001*	1682(15.2%)	1615(14.6%)	1466(13.4%)	<0.001*
Alcohol	1013(8.6%)	1156(9.9%)	1080(9.2%)	0.006*	1204(10.3%)	1037(8.8%)	1008(8.6%)	<0.001*	1208(10.3%)	1079(9.2%)	962(8.2%)	<0.001*
Hypertension	2920(24.9%)	3005(25.6%)	3005(25.6%)	0.347	3076(26.2%)	3004(25.6%)	2850(24.3%)	0.002*	3076(26.2%)	3031(25.8%)	2823(24.1%)	<0.001*
Stroke	154(1.3%)	174(1.5%)	164(1.4%)	0.540	171(1.5%)	172(1.5%)	149(1.3%)	0.351	192(1.6%)	153(1.3%)	147(1.3%)	0.025*
Diabetes	1232(10.5%)	1351(11.5%)	1355(11.5%)	0.016*	1344(11.5%)	1345(11.5%)	1249(10.6%)	0.073	1359(11.6%)	1354(11.5%)	1225(10.4%)	0.007*
Depression	428(3.6%)	464(4.0%)	409(3.5%)	0.154	404(3.4%)	439(3.7%)	458(3.9%)	0.170	405(3.5%)	438(3.7%)	458(3.9%)	0.181

*: P-value<0.05

Table S2 The associations between tertiles of air pollutant concentrations and MCI in multi-pollutant and interaction models.

Variable	Crude OR	Model 1#	Model 2##	Model 3###
$PM_{2.5}$ Tertile				
T1	Ref.	Ref.	Ref.	Ref.
T2	0.95 (0.88 - 1.03)	0.92 (0.82 - 1.03)	0.95 (0.82–1.11)	0.58 (0.45–0.74)*
T3	1.19 (1.10 - 1.29)*	1.23 (1.11 - 1.37)*	2.00 (1.61–2.50)*	1.74 (1.27–2.37)*
PM_{10} Tertile				
T1	Ref.	Ref.	Ref.	Ref.
T2	1.21 (1.12 - 1.31)*	1.19 (1.06 - 1.33)*	1.19 (1.02–1.37)*	1.33 (1.14–1.56)*
T3	1.16 (1.07 - 1.26)*	1.10 (0.98 - 1.23)	0.66 (0.51–0.85)*	0.76 (0.58–0.99)*
SO_2 Tertile				
T1	Ref.	Ref.	Ref.	Ref.
T2	1.18 (1.09 - 1.28)*	1.25 (1.11 - 1.40)*	1.42 (1.25–1.61)*	1.06 (0.87–1.28)
T3	1.18 (1.09 - 1.28)*	1.20 (1.07 - 1.34)*	1.61 (1.36–1.91)*	1.21 (0.96–1.54)
O_3 Tertile				
T1	Ref.	Ref.	Ref.	Ref.
T2	1.19 (1.10 - 1.28)*	1.13 (1.02 - 1.27)*	0.93 (0.82–1.06)	0.97 (0.85–1.11)
T3	0.88 (0.81 - 0.95)*	0.85 (0.75 - 0.95)*	0.67 (0.57–0.78)*	0.67 (0.58–0.79)*
CO Tertile				
T1	Ref.	Ref.	Ref.	Ref.
T2	0.91 (0.84 - 0.98)*	0.88 (0.79 - 0.98)*	0.70 (0.60–0.82)*	0.66 (0.56–0.78)*
T3	0.86 (0.80 - 0.94)*	0.93 (0.83 - 1.03)	0.66 (0.53–0.82)*	0.66 (0.53–0.82)*
NO_2 Tertile				
T1	Ref.	Ref.	Ref.	Ref.
T2	1.07 (0.99 - 1.16)	1.09 (0.97 - 1.22)	0.97 (0.81–1.16)	1.07 (0.88–1.29)
T3	0.95 (0.88 - 1.03)	1.04 (0.93 - 1.16)	0.89 (0.70–1.13)	0.99 (0.77–1.26)

#: **Model 1** adjusted for gender, age, stroke, monthly income, BMI, exercise habits, Married/Cohabitant, Smoking, Alcohol, Hypertension, and Diabetes.

##: **Model 2** adjusted for gender, age, stroke, monthly income, BMI, exercise habits, Married/Cohabitant, Smoking, Alcohol, Hypertension, Diabetes, and co-pollutants.

###: **Model 3** adjusted for gender, age, stroke, monthly income, BMI, exercise habits, Married/Cohabitant, Smoking, Alcohol, Hypertension, Diabetes, co-pollutants, and $PM_{2.5} \times SO_2$ interaction.

*: P-value<0.05

Table S3 Sensitivity analysis of the associations between air pollutants and MCI, additionally adjusted for depression.

Variable	Crude OR	Model 1#	Model 2##	Model 3###
$PM_{2.5}$ Tertile				
T1	Ref.	Ref.	Ref.	Ref.
T2	0.95 (0.88 - 1.03)	0.94 (0.84–1.06)	0.95 (0.82–1.11)	0.58 (0.45–0.74)*
T3	1.19 (1.10 - 1.29)*	1.30 (1.17–1.45)*	2.01 (1.61–2.50)*	1.75 (1.28–2.38)*
PM_{10} Tertile				
T1	Ref.	Ref.	Ref.	Ref.
T2	1.21 (1.12 - 1.31)*	1.22 (1.09–1.37)*	1.18 (1.02–1.37)*	1.33 (1.14–1.56)*
T3	1.16 (1.07 - 1.26)*	1.16 (1.04–1.30)*	0.66 (0.51–0.85)*	0.76 (0.58–0.99)*
SO_2 Tertile				
T1	Ref.	Ref.	Ref.	Ref.
T2	1.18 (1.09 - 1.28)*	1.29 (1.16–1.45)*	1.42 (1.25–1.61)*	1.06 (0.87–1.28)
T3	1.18 (1.09 - 1.28)*	1.25 (1.11–1.40)*	1.61 (1.36–1.92)*	1.21 (0.96–1.54)
O_3 Tertile				
T1	Ref.	Ref.	Ref.	Ref.
T2	1.19 (1.10 - 1.28)*	1.14 (1.02–1.27)*	0.93 (0.81–1.06)	0.97 (0.85–1.10)
T3	0.88 (0.81 - 0.95)*	0.85 (0.76–0.95)*	0.67 (0.57–0.78)*	0.67 (0.58–0.79)*
CO Tertile				
T1	Ref.	Ref.	Ref.	Ref.
T2	0.91 (0.84 - 0.98)*	0.91 (0.81–1.01)	0.70 (0.60–0.82)*	0.66 (0.56–0.78)*
T3	0.86 (0.80 - 0.94)*	0.93 (0.83–1.04)	0.66 (0.53–0.82)*	0.66 (0.53–0.82)*
NO_2 Tertile				
T1	Ref.	Ref.	Ref.	Ref.
T2	1.07 (0.99 - 1.16)	1.12 (1.00–1.25)*	0.97 (0.81–1.16)	1.07 (0.88–1.29)
T3	0.95 (0.88 - 1.03)	1.04 (0.93–1.16)	0.89 (0.70–1.13)	0.99 (0.77–1.26)

#: **Model 1** adjusted for gender, age, stroke, monthly income, BMI, exercise habits, Married/Cohabitant, Smoking, Alcohol, Hypertension, Diabetes and Depression.
##: **Model 2** adjusted for gender, age, stroke, monthly income, BMI, exercise habits, Married/Cohabitant, Smoking, Alcohol, Hypertension, Diabetes, Depression, and co-pollutants.
###: **Model 3** adjusted for gender, age, stroke, monthly income, BMI, exercise habits, Married/Cohabitant, Smoking, Alcohol, Hypertension, Diabetes, Depression, co-pollutants, and $PM_{2.5} \times SO_2$ interaction.
*: P-value<0.05

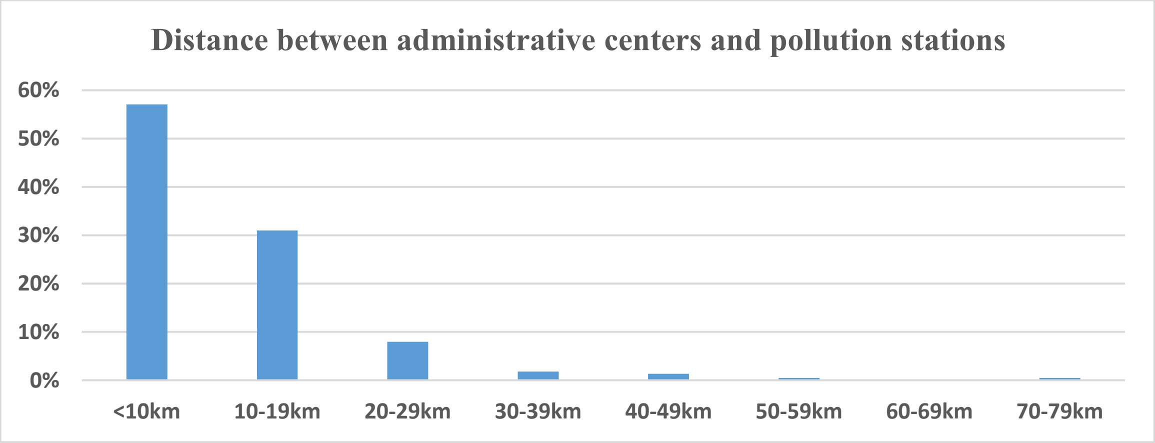

Figure S1 Distance between administrative centers and pollution stations
The bar chart illustrates the distribution of administrative centers categorized by their distance to the nearest monitoring station. The X-axis represents the distance intervals (in kilometers), and the Y-axis displays the percentage of administrative centers within each distance category.
